# A rare case of PLA2R- and THSD7A-positive idiopathic membranous nephropathy

**DOI:** 10.1590/2175-8239-JBN-2019-0077

**Published:** 2019-10-28

**Authors:** David Campos Wanderley, Bárbara Dornelas Jones, Fabricio Augusto Marques Barbosa, Stanley de Almeida Araujo

**Affiliations:** 1 Instituto de Nefropatologia, Belo Horizonte, MG, Brasil.; 2 Universidade Federal de Minas Gerais, Centro de Microscopia Eletrônica, Belo Horizonte, MG, Brasil.; 3 Hospital Evangélico, Belo Horizonte, MG, Brasil.; 4 Hospital UNIMED, Belo Horizonte, MG, Brasil.

**Keywords:** Glomerulonephritis, Membranous, Receptors, Phospholipase A2, Thrombospondin 1, Glomerulonephritis, IGA, Glomerulonefrite Membranosa, Receptores da Fosfolipase A2, Trombospondina 1, Glomerulonefrite por IgA

## Abstract

Idiopathic membranous nephropathy (IMN) is a frequent cause of nephrotic syndrome in adults. In terms of etiology, the condition may be categorized as primary/idiopathic or secondary. Literature on the pathophysiology of IMN has indicated the presence of autoantibodies (PLA2R and THSD7A) directed against podocyte antigens. The detection of antibodies against a domain favors IMN. The presence of autoantibodies against one of the domains would in theory exclude the possibility of there being autoantibodies against the other domain. However, cases of patients with PLA2R- and THSD7A-positive disease have been recently reported, showing that antibodies against two targets may be concomitantly produced via yet unknown pathophysiological mechanisms. This study reports the case of a 46-year-old male patient with nephrotic-range proteinuria, hematuria, hypoalbuminemia, and hypercholesterolemia submitted to biopsy and histopathology examination (LM, IF, IHC, and EM) eventually diagnosed with PLA2R- and THSD7A-positive IMN associated with IgA nephropathy, stressing our experience with the use of IgG subclasses, PLA2R, and THSD7A in the workup for MN and the relevance of adopting a broad and adequate approach to elucidating and acquiring knowledge of the pathophysiology of IMN.

## RESUMO

A Nefropatia Membranosa Idiopática (NMi) é uma frequente causa de síndrome nefrótica em adultos e sua etiologia pode ser estratificada em primária/idiopática ou secundária. O conhecimento da fisiopatologia da NMi sugeriu a presença de autoanticorpos (PLA2R e a THSD7A) direcionados contra antígenos existentes nos podócitos. A detecção de anticorpos contra um domínio favorece NMi. A presença de autoanticorpos contra um desses domínios autoexcluiria a possibilidade de autoanticorpos contra o outro domínio; no entanto, recentemente foram descritos casos que apresentaram dupla positividade para PLA2R e THSD7A, comprovando que, por mecanismos fisiopatológicos ainda não conhecidos, raramente pode existir produção concomitante de anticorpos contra os dois alvos. O presente estudo tem por objetivo relatar o caso de um paciente de 46 anos de idade, do sexo masculino, que apresentou quadro de proteinúria nefrótica, hematúria, hipoalbuminemia e hipercolesterolemia submetido a biópsia e exame histopatológico (ML, IF, IHQ e ME), confirmando um caso raro de NMi com positividade dupla para os anticorpos anti-PLA2R e anti-THSD7A e associação à nefropatia por IgA, mostrando nossa experiência com a utilização de subclasses de IgG, PLA2R e THSD7A na rotina laboratorial para a investigação da GNM e enfatizando a importância de uma abordagem ampla para adequada elucidação e conhecimento dos mecanismos fisiopatológicos na NMi.

## INTRODUCTION

Membranous nephropathy (MN) is a frequent cause of nephrotic syndrome in adults. In terms of etiology, the condition may be categorized as primary/idiopathic (IMN) or secondary (SMN). Since clinical, biochemical, morphologic, and immunophenotypic traits are nonspecific in most cases, patients require testing for a number of conditions associated with secondary forms of the disease, including malignant tumors, infectious diseases, autoimmune diseases, and drug abuse. Therefore, the diagnosis of primary forms of the disease can only be established after all known secondary causes have been ruled out.^1^

Literature on the pathophysiology of IMN has indicated the presence of autoantibodies directed against podocyte antigens. The ensuing formation of immune deposits in the subepithelial space alters podocyte disposition and organization, thus disrupting the polarity of the glomerular basement membrane (GBM) and culminating with proteinuria.^2^ M-type phospholipase A2 receptor (PLA2R) was the first recognized antigen, followed by thrombospondin type-1 domain-containing 7A (THSD7A). Identified in more than 70% of the cases of IMN, PLA2R has been considered the main antigen in membranous nephropathy, although it is often absent in secondary forms of the disease and other forms of glomerulopathy.^3^^-^ 5 Antibodies against THSD7A have been observed in approximately 10% of the PLA2R-negative patients with IMN.

Therefore, PLA2R and THSD7A have been identified as the two main targets of autoantibodies in IMN, wherein the presence of antibodies against one domain would in theory rule out the presence of autoantibodies against the other domain.^6^^-^ 8 However, cases of patients with PLA2R- and THSD7A-positive disease have been recently reported, showing that antibodies against two targets may be concomitantly produced via yet unknown pathophysiological mechanisms.^4^

This study reports the clinical and histopathology findings acquired via light microscopy (LM), immunofluorescence (IF), immunohistochemistry (IHC), and electron microscopy (EM) of a rare case of PLA2R- and THSD7A-positive IMN.

## CASE REPORT

**Figure 1. A, B, and C:**
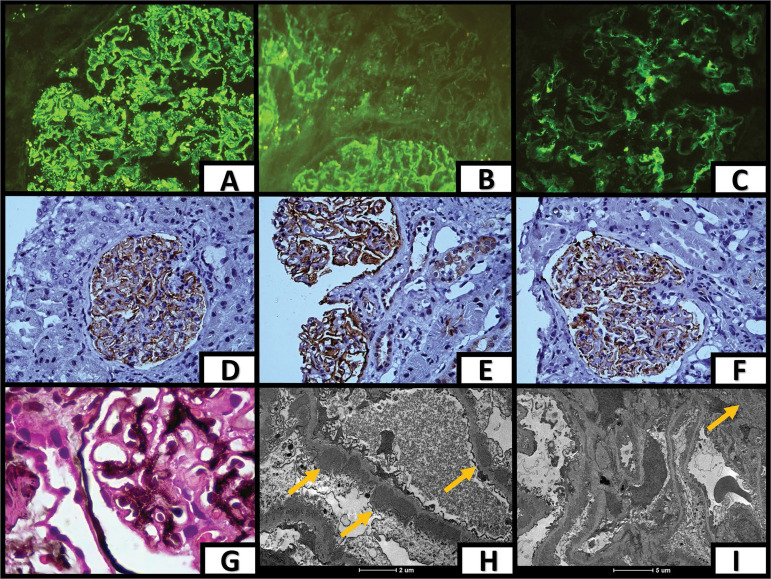
immunohistochemistry staining - diffuse IgG and C3 granular deposits along the GBM and granular IgA deposits in the mesangium, respectively; **D, E, and F:** immunohistochemistry staining - strong staining for de forte PLA2R, THSD7A, and IgG4 along the GBM, respectively; **G:** oil immersion light microscopy image of a specimen stained with Jones methenamine silver - thickened GBM with spikes; **H and I:** electron microscopy image - subepithelial, mesangial/paramesangial deposits along the GBM - see arrows.* *GBM - glomerular basement membrane*.

A 46-year-old male was admitted to a hospital in the Metropolitan Area of Belo Horizonte, Minas Gerais, Brazil, with leg edema progressing since six months prior to hospitalization, associated with foamy urine and weight gain of 10 Kg. The patient had a history of systemic hypertension, hyperuricemia, dyslipidemia, and recurring use of non-steroid anti-inflammatory drugs. Physical examination revealed he had edema on both legs (2+/4+). His blood pressure was normal (BP 120x60 mmHg) and he breathed normally in ambient air. The patient was on amoxicillin/clavulanic acid for community-acquired pneumonia. His renal function was preserved (serum creatinine: 0.86 mg/dL) and he did not have fluid and electrolyte disorders or anemia (Hb: 12.1 mg/dL). Urine tests showed urinary protein (3+/3+) and hematuria with erythrocyte dysmorphism (30%), 4.0 g of urinary protein over 24 hours, hypoalbuminemia (albumin: 1.5 mg/dL), and hypercholesterolemia (total cholesterol: 217 mg/dL). Serology tests for HIV, hepatitis B and C, VDRL, ANA, RF, and ANCA were negative. C4 level: 35.5 mg/dL; C3 level: 126.3 mg/dL. His transthoracic echocardiogram showed an ejection fraction of 65% without ventricular dysfunction, while venous ultrasound examination of the legs did not reveal signs of deep venous thrombosis. The patient was suspected for glomerular disease, and a kidney biopsy was thus ordered. Examination of biopsy specimens on a light microscope showed glomeruli with a slightly expanded mesangial matrix and increased mesangial hypercellularity, a diffusely thickened GBM with small spikes (Figure 1-G), and preserved tubulointerstitial and vascular spaces. Immunohistochemistry analysis with fluorescein revealed a speckled pattern along the basement membrane stained positive for IgG, C3, Kappa and Lambda. Mesangial expression of IgA, C3, Kappa and Lambda was also found (Figures 1-A to 1-C). Peroxidase immunohistochemistry showed a strong granular pattern along the basement membrane stained positive for IgG1, IgG4, THSD7A, and PLA2R (Figures 1-D to 1-F). Examination with an electron microscope helped to identify electron-dense subepithelial and mesangial immune deposits with diffuse podocyte foot process alterations, including flattening and effacement (Figures 1-H and 1-I). These alterations are consistent with stage II membranous nephropathy associated with IgA nephropathy stained positive for PLA2R and THSD7A. The patient underwent examination with upper gastrointestinal endoscopy, colonoscopy, and chest/abdomen computed tomography scans, and was tested for serum CEA and CA 19-9, but relevant alterations were not found. The patient is stable and has had steady decreases in urine protein levels (3.5 grams/24 hours after two months). He is currently on dual RAAS blockade, hemodynamically stable, and free of renal function alterations.

## DISCUSSION

IMN produces a wide range of clinical manifestations with different progression patterns, some resulting in spontaneous remission of proteinuria (with excellent long-term prognosis) and others in persistent nephrotic syndrome (culminating with end-stage renal disease).^7^^,^ 8 Our patient had the classic signs and symptoms of nephrotic syndrome - hypercholesterolemia, hypoalbuminemia, edema, and proteinúria (4 grams/24 hours) - along with hematuria and erythrocyte dysmorphism, without renal function alteration.

Patients with nephrotic syndrome must be analyzed for the most prevalent secondary causes of disease, for which our patient was negative. Renal biopsy ordered with diagnostic intent revealed a diffusely thickened GBM with spikes, in addition to a speckled pattern along the basement membrane stained positive with fluorescein for IgG, C3, Kappa and Lambda, confirming the diagnosis of IMN.^5^ Strong mesangial expression of IgA was also described, thus corroborating an association with IgA nephropathy.^9^ Examination with an electron microscope may show subepithelial immune deposits and foot process effacement^5^ and support the staging of the disease, as in the case reported.

Despite the support provided by extensive workup, ruling out secondary causes of MN is still a challenge for physicians. This is why the last four decades have seen the introduction of supplementary methods to dichotomize cases and improve the discrimination of individuals with IMN from subjects with SMN, via qualitative differentiation of IgG fractions,^10^^-^ 12 electron microscopy, PLA2R^2^^,^ 3 ^,^ 5 and THSD7A^7^^,^ 8 serum levels and histology. Circulating antibodies against PLA2R and THSD7A, which mainly belong to the IgG4 subclass known to be present in the early stages of disease, rank among the most recently studied methods. Immunohistochemistry analysis of our patient showed a strong granular pattern along the basement membrane stained positive for PLA2R, THSD7A, and IgG4, suggesting a diagnosis of IMN.

However, positive tests for PLA2R and THSD7A as seen in our patient were believed to be mutually exclusive.^6^^-^ 8 Only in 2016 the first cases of PLA2R- and THSD7A-positive patients were described in the literature.^4^ A recent meta analysis indicated that only six cases of PLA2R- and THSD7A-positive patients had been published until then.^13^ Ours is, therefore, the seventh reported case, with the addition that our patient possibly has associated IgA nephropathy.

Despite the significant correlation between PLA2R, THSD7A, and IMN, associations have also been described with malignant tumors, autoimmune disease, hepatitis C, and sarcoidosis.^10^^,^ 14 ^-^ 18 Serum anti-PLA2R antibody levels have also been associated with disease and used as predictors of response to therapy.^8^^,^ 14 Decreased levels have been associated with response to therapy in patients with IMN, generally followed by remission of proteinuria within months.^15^ For purposes of comparison, anti-THSD7A antibody levels do not always correlate with urine protein and may not predict disease remission.^12^^,^ 16

The diagnosis of MN and the definition of the primary and secondary forms of the disease are undergoing significant change, since the physiopathogenic factors connected to the condition are still being clarified. This study presented the use of IgG subclasses, PLA2R, and THSD7A in the workup for the investigation of MN. Unlike previous publications, our report showed that, although rare, PLA2R- and THSD7A-positive cases might occur in an even rarer and possibly unique combination with IgA nephropathy. Our study stressed the importance of adopting a broad investigative approach including light microscopy, immunofluorescence, electron microscopy, and immunohistochemistry to properly elucidate the pathophysiology of IMN.
